# Transcendent Experiences Among Pilgrims to Lourdes: A Qualitative Investigation

**DOI:** 10.1007/s10943-021-01306-6

**Published:** 2021-06-25

**Authors:** Emmylou Rahtz, Sara L. Warber, Sarah Goldingay, Paul Dieppe

**Affiliations:** 1 The European Centre for Environment and Human Health, The University of Exeter Medical School, Royal Cornwall Hospital, Truro, Cornwall, TR1 3HD UK; 2 Department of Family Medicine, University of Michigan Medical School, 1018 Fuller St, Ann Arbor, MI 48104-1213 USA; 3 Department of Drama, University of Exeter, Alexander Building, Thornlea, New North Road, Exeter, EX4 4LA Devon UK; 4 The University of Exeter Medical School, South Cloisters, St Luke’s Campus, Heavitree Road, Exeter, EX1 2LU Devon UK

**Keywords:** Pilgrimage, Well-being, Transcendent experience, Lourdes, Therapeutic landscapes

## Abstract

Millions of pilgrims visit Lourdes each year, often seeking revitalisation rather than miraculous cures. We sought to understand the phenomenon of transcendent experiences. We spoke with 67 pilgrims including assisted pilgrims, young volunteers and medical staff. About two in five reported a transcendent experience: some felt they had communicated or had close contact with a divine presence, while others reported a powerful experience of something intangible and otherworldly. Transcendent experiences are an important feature of pilgrimage to Lourdes and the place offers the faithful a means of connecting with the divine, with nature and with the self.

## Background

Lourdes is a Catholic pilgrimage site in South West France known as a sanctuary for healing. In 1858 a young woman, Bernadette Soubirous, had visions in a grotto outside Lourdes of a woman who identified herself to Soubirous as the Virgin Mary. Soubirous received several instructions, including exhortations that people should come in procession to the grotto, build a chapel there, and drink and bathe in the waters. People began to report miraculous cures as a result of these practices, and there have been thousands of claims of healing in the subsequent years. These are submitted to Lourdes’ own Medical Bureau for investigation: 70 “unexplained healings” have been confirmed as miraculous, out of more than 7000 candidate cases (Lourdes Sanctuary, n.d.).

Lourdes has extensive infrastructure to support the estimated six million pilgrims who visit each year, including 80,000 “assisted pilgrims” who are officially sick or have a disability (Lourdes Sanctuary, n.d.). Most, although not all, are Catholic. At the heart of the site is the Grotto; nearby are basilicas, churches, baths where pilgrims are immersed in the holy water and rows of taps where they can wash their hands and faces in the water, and collect it in bottles. Services and processions take place each day, often outdoors, attended by assisted pilgrims, volunteers, healthcare professionals, priests and visitors. Walkways facilitate the daily processions, which can include thousands of people, including many with severe disabilities. The setting is pleasant, the Grotto being adjacent to the river, with meadows, woodlands and mountains within view. Many assisted pilgrims, volunteers and others come back year after year in groups, with families or alone (Agnew, 2019). The mainstream perception can be that pilgrims visit in the hope of a miracle, a view that is potentially patronising to those who visit regularly (Notermans, 2007). The reality has been markedly different for some decades, with pilgrims, clergy and Lourdes officials adopting a narrative which prioritises emotional and spiritual refreshment (Gesler, 1996; Harris, 2013; Notermans, 2007; Warfield et al., 2014). Gesler’s seminal work on therapeutic landscapes framed Lourdes as a site for healing rather than cure, and it may be deemed a “third space”, away from home and work or home and hospital, thus giving opportunities for emotional refuge (Bell et al., 2018). Pilgrims are likely to travel with expectations but these relate to deepening their faith, experiencing community, and perhaps finding temporary respite from health conditions (Goldingay et al., 2021). Among a large group of pilgrims travelling to Lourdes from the Netherlands, “praying for my own healing” was rated as the least important aspect of the Lourdes experience from a list of 20 items (Pieper & Van Uden, 1994). Research about Lourdes tends to explore broader therapeutic effects rather than miraculous cures (Harris, 2013; Higgins & Hamilton, 2019; Perriam, 2015; Warfield et al., 2014).

Previous research visits to Lourdes by two of the authors brought us to an understanding that pilgrims visit Lourdes for a complex variety of holistic reasons, including to serve others, for their own well-being, and for spiritual renewal. We heard people speak of profound spiritual experiences that resulted in improved well-being and symptomatic relief in the absence of any change in a medical condition. These incidents appeared to be intense and extraordinary. One pilgrim with serious health conditions recounted how “I just felt this warmth go over me […] wash over me, and it just felt like I was being enveloped with it and just almost… like… being held. And it took over my body and I felt just complete calm and peace and the pain just disappeared into insignificance” (Goldingay et al., 2014). We wanted to explore these experiences in more detail, yet the terminology for experiences such as these is problematic and people often have difficulty trying to describe them. In this paper, we use the word “transcendent” to describe the out of the ordinary experiences of connection, understanding, wonder, or knowing described by our participants. “Transcendent experience” is both the most common term and the closest to our specific understanding, although we draw on literature using a range of terms. William James describes “states of insight into depths of truth unplumbed by the discursive intellect. They are illuminations, revelations, full of significance and importance, all inarticulate though they remain; and as a rule they carry with them a curious sense of authority” (James, 1902). Waldron provides a useful overview of different terms in use for these experiences and concludes that “the transcendent experience is a spontaneous, extraordinary state-of-consciousness event which breaks through the existing boundaries of knowledge and experience” (Waldron, 1998). Seidlitz (Seidlitz et al., 2002) and Fisher (Fisher, 2010) both use the word “transcendent”, and Fisher stresses the idea that spiritual well-being is dependent on our relationships with self, others, the environment and “transcendent Others”. Transcendent experiences have been defined and explored in depth by Levin and Steele who reported that while they are difficult to characterise, they typically involve a state of altered consciousness and a sense that there is more to reality than is evident within the usual everyday boundaries (Levin & Steele, 2005), such as a divine presence beyond physical experience. For Stöckigt, a transcendent experience is one which lifts a person beyond the self (Stöckigt et al., 2015), and Alling provides an excellent overview including non-religious examples such as certain intense examples of conversion experiences, romantic love and encounters with nature, concluding that transcendent experiences are mystical phenomena (Alling, 2015). Surveys suggest that a third of people in the USA and the UK have had an intense religious experience like a transcendent experience that “lifted them outside of themselves” (Levin & Steele, 2005).

And yet, although Lourdes is evidently a place where many people deepen their connection with the divine, we were unable to find previous literature specifically exploring transcendent experiences at Lourdes. Given that few pilgrims experience miraculous healing and many may come for a host of other reasons, the purpose of our research was to explore whether pilgrims visiting Lourdes had transcendent experiences and to examine their nature.

## Methods

In June 2017, we spent eight days in Lourdes conducting extensive interviews and focus groups with pilgrims. Ethical approval was granted by the College of Humanities (HUMS) Research Ethics Committee, University of Exeter. The authors comprise a health researcher, a humanities scholar and two medical doctors; all had experience of collecting and analysing qualitative data. In addition, a former nurse assisted our fieldwork. Some of us had visited Lourdes before, both as volunteers and as researchers, others were visiting for the first time. We stayed in the centre of Lourdes, attended several services and processions and visited significant places. We kept detailed ethnographic notes documenting our impressions and the formal and informal conversations and encounters we had, and we discussed these regularly during and after the field trip.

Most participants were part of three large pilgrimage groups: these were English-speaking groups, an important criterion for our research. By prior arrangement, we had timed our field trip to coincide with two of these groups’ visits: a large congregation from London and a British Catholic order. An existing contact within these each group acted as a facilitator and gatekeeper, enabling us to interview other members. During our trip we met the leader of a group from a small European country at an event at the Medical Bureau, who agreed to introduce us to the many English-speaking members of that group. Other participants were pilgrims we met and invited opportunistically to take part; for example, we met several at services and gatherings at the Medical Bureau. Participants were selected to garner input from people with different reasons for visiting Lourdes and with a range across age, gender and role. Each participant gave informed consent; in addition, group leaders, as legally authorised representatives, gave consent for school-aged participants to contribute. Some participants were interviewed individually, others in pairs or groups: discussions in these different contexts can produce different meanings and perceived expectations, and we have therefore specified the interview method with each quote. All interviews were conducted face to face and were recorded. Interviews took place in opportune and sometimes natural settings, including benches by the river, hotel gardens, hospital beds and the Medical Bureau.

**Fig. 1:**
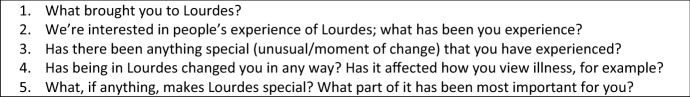
Interview schedule

Figure 1 shows our semi-structured interview schedule; these core questions were supplemented with follow-up questions. We asked whether people had experienced anything “anything special (unusual/moment of change)” as a colloquial alternative to the term “transcendent”.

Interviews lasted between ten minutes and one hour. All interviews were transcribed and our own notes typed up. We used Atlas.ti software to assist the analysis. This thematic analysis (Braun & Clarke, 2006) focuses on people’s unusual or transcendent experiences. ER read all interviews: her notes of recurring concepts formed an initial list of inductive codes. These were supplemented with a list of a priori codes derived from our research questions, including items around transcendent experiences. ER coded eight interviews with this first code frame, and refined the code frame after extensive team discussions. SLW coded two interviews to check for consistency: we found a high level of consensus in the coding, and made further adjustments to the codes and how they were applied as a result. The full data set was then coded with this refined code frame. Further discussions among the whole team led to the identification of a number of themes: we decided to focus on the transcendent experiences that had been an a priori interest. We explored the data further for instances where people described the expectations, experiences, conversations and contexts around transcendent experiences.

Participants are identified by pseudonyms, age and gender, apart from those who participated in focus groups where it was not possible to know who was speaking.

## Results

We conducted interviews (36 people), paired interviews (six people) and focus groups (25 people across five groups; four involved teenaged school students from the London congregation, one involved young adults associated with the British Catholic order), giving a total of 67 participants. As shown in Table 1, ages ranged from 15 to 87: half were aged under 25 and a fifth were 75 or older. More than two-thirds of participants were female, a figure in line with the gender distribution typically seen among pilgrims (Harris, 2013).

**Table 1:** Demographic profile of all study participants

	*N*	%
Total	67	100%
*Age*		
Under 18	20	30%
18–24	14	21%
25–34	5	7%
35–44	3	4%
45–54	2	3%
55–64	5	7%
65–74	5	7%
75+	13	19%
*Gender*		
Female	48	72%
Male	19	28%

Whilst we did not record ethnicity, participants were diverse in profile with particular diversity evident in the London congregation. Participants included assisted pilgrims, volunteers (including teenaged school students), healthcare professionals, members of the clergy and family members travelling with assisted pilgrims. No one refused to take part; however, we were unable to interview some pilgrims due to the groups’ busy schedules.

Approximately 26 people described a transcendent experience on some level; the figure is approximate because about ten of these accounts came from focus groups, and therefore cannot always be definitively linked with an individual. Four of the five focus groups discussed transcendent experiences, with one, Student focus group 1, going into some detail, as is reflected in the quotes that follow. The profile of participants reporting transcendent experiences was broadly similar to that of all participants. Transcendent experiences were judged by the research team on the content of interviews, rather than on a “yes” response to the question about “something special”.

Two main themes were identified from the interviews around transcendent experiences. “Communicating with the Virgin Mary and God” documents people’s sense of close connection with the divine, often at the Grotto. “Something otherworldly, non-ordinary” described people’s transcendent experiences of something intangible and transcendental.

### Theme 1: Communicating with the Virgin Mary and God—“I Feel that I am Nearer the Virgin Mother”

This superordinate theme encompasses several different sensory relationships with the divine, identified as God or, more often, as the Virgin Mary. These are a sense of feeling physically close to the divine; a sense of direct and responsive communication; and other experiences: there is overlap between the categories. These experiences are associated with the peacefulness of the place, as well as powerful positive feelings or intense emotions that include crying or tearfulness (described in the section “What follows a transcendent experience”). Participants felt that although “God is everywhere”, at Lourdes they could really relate and have quality time. For example:

Susan (female, 35): And I feel I can really relate with the heavens above. I know that God is everywhere, but being here gives me some very good ‘me time’… quality time with God.

#### Feeling Physically Close to the Divine

One of the most common experiences was that of feeling physically close to God or the Virgin Mary. People felt that the Virgin Mary was with them, watching over them, and this closeness was a source of reassurance. Michaela (female, 61) was interviewed with her husband and explained how “with the holy rosary from here in my hand I felt that Our Lady was with us, and it would be OK”, and went on to describe a sense of closeness.

For many, the Grotto was focal to their transcendent experience, and was particularly associated with the Virgin Mary. As one student said, “it’s where Bernadette saw Mary so it’s obviously a significant place to be. People go there and feel closer to Mary and closer to God as well” (Student focus group 4). For one woman, feeling aware of the many other people at the Grotto and hearing their singing helped her to feel closer to the Virgin Mary:

Pia (female, 59): While I am at the Grotto, and especially if there are a lot of people, and if there is singing, hymns… then I feel that I am more nearer the Virgin Mother.

The Grotto could offer contrasting atmospheres that never the less led people to similar experiences of feeling connected with something divine. A young man described the power of the Grotto but through a markedly different experience from Pia’s. The silence and calm at the Grotto, especially at night, allowed him to connect with the Virgin Mary, who he felt was watching over him:

Kevin (male, 24): What I found at the Grotto was pure silence… I can’t say for sure that I can feel Mary, but I feel that someone, and that probably is Mary, is there with us… it’s so still and it’s so peaceful… someone is watching and someone is saying it’s going to be all right… the Grotto is the most special part – especially at night-time… with the stillness then that’s when... you know they are watching over us.

He went on to describe the Grotto’s natural shape and the sound of the river, emphasising the importance of nature. James (male, 25) told a similar story while talking about his relationship with the Virgin Mary, wherein the peacefulness of the Grotto gave rise to a sense of solitude whilst also making him feel connected with the reassuring presence of the divine in a profound way. The sense of therapeutic understanding he describes reflects his experience a divine presence.

James: The Grotto… There is something there that I cannot explain, cannot articulate… especially during the night when it is empty… I have had the opportunity to just be there by myself and that’s just completely… I just cannot explain what that’s like… you can pour out every feeling that you have… and there is a sense of understanding there.

This sense of peace was described in many of the accounts and was often a key aspect of a transcendent experience, particularly those taking place at the Grotto. Some described it as peace inside themselves, and for others it reflected the peace of the environment. Some groups engaged in regular “night prayers”, visiting the Grotto in smaller groups at night, and some individuals had adopted their own practices of this kind. The natural aspects of the Grotto were often mentioned, including the cave itself, as well as the river beside it and the trees and fields beyond. A group leader also felt closer to God and attributed this to differences within himself whilst he was in Lourdes:

Mark (male, 35): You’re in the presence of Jesus, as you would be at any mass but… it’s not God that is different – it’s you. You are in a different context of yourself really. And you’re experiencing God.

Some felt touched or embraced by the Virgin Mary. One of the school students said: “So, I came and I prayed to Mary and then she touched me a lot and I felt like I was actually talking to her.” (Student focus group 1). Michaela (female, 61, interviewed in a pair) described how: “every time you come you feel that Our Lady is embracing you.” Another woman felt touched, and her account also links with Theme 2, the idea that there was “something there”:

Maria (female, 43): The first time when I came around the sanctuary in the Grotto, there’s something in there. I can’t help it. I was just crying. I couldn’t help it and it makes you feel like you’d been touched. Until now I can remember – it’s really vivid.

The experience was very real to her, and she found it emotional and memorable. Several others talked about crying during a transcendent experience, and some became tearful while recounting it.

#### Being in Conversation with the Divine

A related experience was that of feeling in direct conversation with the Virgin Mary or God, especially when praying at the Grotto. A group of school students described clear experiences of this kind during a focus group. One said “Like, yesterday I was communicating with Mary and I felt so good. So good.” Another had had a similar experience: “It was like we were communicating with our Mother, Mary. Telling her your problems and it looked like she is talking to you.” A third echoed this: “yesterday I felt so close – like it was so satisfying to confess directly to God.” (Student focus group 1). This group talked at some length about their experiences at the Grotto and echoed each other’s accounts, which were reminiscent of being in a confessional, or of talking therapy.

This was reiterated by two young men in individual interviews. For Kevin (male, 24), a divine presence was with him at the Grotto, listening to his worries in a therapeutic manner: “It just feels like someone is there and someone is listening to you and then that’s always reassuring”. John (male, 26) felt that Lourdes provided special conditions for speaking with God: “And, whilst God – you can speak to God everywhere – I feel that I’m much closer to my faith here.” In these accounts, and others, prayer was a route to deep connection with the divine.

One participant felt that he had heard the Virgin Mary speak to him in a very literal sense. Sean, who had converted to Catholicism late in life, had a transcendent experience that guided him to Lourdes:

Sean (male, 84): And, I looked up at Our Lady and I said, can you tell me please what you want me to do? And, now, people scoffed at this. There was a voice in my ear, ‘why don’t you come and see me in Lourdes?’ And, I think that was the one real experience I’ve had.

This did not take place at Lourdes, but the communication was, by Sean’s account, the reason for him coming to Lourdes; as though the Virgin Mary had called to him from Lourdes. While some accounts of transcendent experiences related to the pilgrims’ current trip, several, including Luke’s, had taken place years before, suggesting that they had a lasting impact whose effects continued to be felt. Other accounts suggest a recurring experience when visiting the Grotto.

#### Other Ways of Connecting with the Divine

As well as these experiences of physical and aural “contact” with the Virgin Mary and God, there were other variations on the idea of connecting with the divine. One woman felt guided in a way that affected her profoundly:

Pauline (female, 49): I think there’s that profound, spiritual experience… Especially, if God sees that perhaps you are not going the way that he wants you to go. He will get you back.

A doctor, Cerys (female, 61), talked about caring for a priest who, through Lourdes, “had thought deeply and therefore met his death very knowingly.” While this account was not explicitly about connecting with God, the idea of “knowing” death gives a sense of the transcendent, and for a priest, meeting death would likely be a gateway to meeting God and communicating with the divine.

A school student described the experience of feeling God within oneself: “You know like they say God’s in everyone, when like you’re ill or something, you can actually like feel him in you” (Student focus group 2). An older woman felt that the Virgin Mary was a constant and powerful presence: “The Madonna is my helper—she helps me every day – my first daughter is with me because I love the Madonna. That is the truth of how I feel.” (Margarita, female, 87).

### Theme 2: Something Otherworldly, Non-Ordinary—“I Cannot Explain, It is Something Really Heavenly”

Pilgrims often described a transcendent experience in Lourdes which involved something significant and out of the ordinary, but they could not define it. This inability to put the experience into words is a uniting characteristic within this theme. Susan (female, 35) said: “You really have to have the experience yourself, I cannot explain, it is something really heavenly… just heavenly”. Maria (female, 43) discussed both her own and her teenaged son’s experiences in these terms: “[my son] said there’s really something in there” and later she added “I don’t want to over-analyse or intellectualise my faith, but there is something there.” Martha (female, 78) talked about praying “very hard with your heart” at the Grotto, and said: “Well, when you remember that the Mother Mary was there, where you are, well it is something else… when I look at [the Virgin Mary], something happens”.

Pauline was adamant about this powerful but imperceptible quality which she, like many others, had experienced at the Grotto:

Pauline (female, 49): There are things that you cannot see but it’s right here – but you can’t see the spiritual, can you? Right, so what you cannot see is affecting you so much.

Jeremy (male, 18) said: “to try and describe what I feel in the Grotto… the feeling is totally intangible”. Luke (male, 79) echoed this in describing the waters at Lourdes, saying that “the water itself has power… It’s really difficult to say because it’s so intangible.” This was a recurring theme throughout the data, and it arose in relation to the uniqueness of Lourdes generally, not just to transcendent experiences. People repeatedly said that there was *something* about Lourdes, something “different”, “heavenly”, “magical” or “special”, but they struggled to define it further. It was something that had to be experienced to be understood; several said that only others who had been to Lourdes could understand. However, it was interesting that although many people told us that they struggled to define their experiences, as though a core part of the experience is that it should be intangible, they described the feelings it gave rise to in detail.

A slightly different perspective emerged of Lourdes feeling qualitatively different from other places. The atmosphere could give the place an otherworldly feeling, where participants were temporarily removed from the everyday world and were closer to a higher force. Kevin stopped just short of saying that the sanctuary was in a different world:

Kevin (male, 24): There are certain places in the domain where it’s a holy area… there’s stillness, there’s no noise… sometimes you’re not in the same – not necessarily world – but you’re not in the same area as everyone else in the hustle and bustle of the town.

Susan (female, 35) said: “I don’t know, it is like something out of this world.” Participants could feel that in Lourdes they were closer to heaven, as described by Lizzie (female, 22, focus group 5—British Catholic order): “the barrier between heaven and here [is] really, really thin at Lourdes.”

Two people had transcendent experiences in which the sun provided key evidence that something significant had taken place. A young doctor interpreted the sudden appearance of the sun as a powerful communication, a confirmation of his beliefs:

John (male, 26): “I’ve had religious experience at the Grotto, which sounds a bit bizarre from a highly educated scientist… I remember making my way to the Grotto – my relationship with God and Mary particularly in Lourdes is very personal... So, I have a conversation, I basically swore straight up, you know, my Grandad’s gone up there [to heaven] so I’ve come to…

Interviewer: Sort you out?

John: Yes [laughs]! Sort you out! And… at that moment just like that [snaps fingers] there was a sudden break and the sun came out and came up straight across the Grotto and then stayed out after that, and when I spoke to other people there, it’s the first time that it had come out in about two weeks. So, reasoning for instance, maybe?

Interviewer: But you thought not?

John: I thought not.

Interviewer: But what did you feel?

John: Affirmation... Affirmation that it wasn’t all a load of ‘holy baloney’.

Like Kevin, John hesitated to call this a divine or supernatural sign, yet it was a highly significant and somewhat mystical moment. Meanwhile an assisted pilgrim had an intense and otherworldly transcendent experience while at the baths. Susan was the only participant to describe the baths, which only a finite number of people can experience, whereas most visitors to Lourdes can visit the Grotto if they wish. Susan felt as though the sun had come out, even though it was a rainy day:

Susan (female, 35): So usually it is raining when I go to the baths, but even though it was raining, on that particular day, I felt as if the sun was coming out for me, with a bright big smile, so I have been wanting to share this, it was one of the happiest days of my life. The heavens were ecstatic because I was doing the right thing. Never mind the cold and the rain outside, I think the sun was shining down. A huge experience [laughs].

These two accounts suggest a connection with the divine through nature and natural phenomena.

### What Follows a Transcendent Experience?

The aftereffects of a transcendent experience varied considerably, from feeling positive emotions, to being drawn back to Lourdes, to a transformative life change. One of the school students reflected simply of her transcendent experience that it “feels nice” (Student focus group 1), and for Margarita (female, 87) it was “a nice experience”. These mild descriptions suggest relatively commonplace experiences. Maria (female, 43) who had described her teenaged son’s transcendent experience, described the gratitude it engendered, saying “it made him realise how lucky he is to have parents with faith” and said he had returned to Lourdes several times as a consequence.

People who felt they had been in conversation with the divine (Theme 1) often recounted powerful positive feelings which they emphasised in their descriptions, e.g. “I felt *so* good”, “it was *so* satisfying”, “*always* reassuring”, “*much* closer” (italics ours). For one of the students who felt that she had talked to Mary, the experience impelled her to return, and to change her focus at Lourdes:

Student focus group 1: I was compelled to come again. But… I was kind of focusing on myself last year. So, it was… to try something that was different and to help other people. And it’s been really satisfying.

As quoted earlier, John (male, 26), felt affirmation of his faith after he experienced a transcendent event, and Susan (female, 35) described “one of the happiest days of my life”. A couple began by describing the happiness and peace they felt after a transcendent experience:

Thomas (male, 63): And it makes us feel happy to be here

Michaela (female, 61): And you feel serenity

However, they went on to describe more consequential effects, where they attributed health changes to the Virgin Mary’s intercession, via the transcendent:

Michaela: … it helped Thomas last year, and my son, when he was ill, she helps us…

Thomas: And the boy…

Michaela: Yes, with our son, she did a very great job for him with his operation… that was her, Our Lady, who is our mother, and as a mother she cannot see suffering of her children.

For them, the Virgin Mary had great powers at the same time as seeming relatable, “our mother”. Although participants talked about both God and the Virgin Mary, the Virgin Mary often had a particularly intimate and empathetic role.

For Pauline, who had a “profound spiritual experience”, the incident left a clear impression that her life would never be the same again. She described this change emphatically:

Pauline (female, 49): And, when you go back your life can NEVER be the same! Never be the same! I believe that wholeheartedly! It can never be the same! That changes your whole life. The whole track that you’re on.

However, as described above, Pauline, like most of the participants, could not quite articulate what the profound spiritual experience had been. She could talk about its intensity and impact, but struggled to define the experience itself.

## Discussion

Among the 67 Lourdes pilgrims we interviewed, about two in five reported an intense spiritual experience that we classified as transcendent, and which was outside the normal range of daily experiences. In accordance with our research question, many of these experiences involved a sense of closeness or a communicative relationship with the divine. Many identified something intangible or undefinable about Lourdes, especially in reference to transcendent experiences. The experiences often had powerful effects on the participants, with some reporting strong emotional responses such as intense joy or tears. Assisted pilgrims, volunteers and others all reported experiences of this nature. In this discussion, we first situate the transcendent experiences in relation to other authors’ accounts and in their effects on the lives of those who experience them. Then, drawing on literature on therapeutic landscapes, we explore the significance of place, referring to Lourdes in general and the Grotto in particular.

### Understanding Transcendent Experiences: Their Intensity and Effects

There were apparent distinctions in the intensity of the transcendent experiences described by our participants. The interviews begin to suggest a spectrum, especially when compared with experiences described by Gesler, Waldron, and Underwood and Teresi. This spectrum would range in three broad gradations from more minor or daily transcendent experiences, via the transcendent experiences that make up the bulk of our observations, and peaking, hypothetically, with the miraculous, which our participants had not experienced.

The less dramatic transcendent experiences described to us share common ground with the “daily spiritual experiences” described by Underwood and Teresi which include feeling the presence of God and feelings of deep inner peace, and which are associated with improvements in quality of life and psychosocial status (Underwood & Teresi, 2002). For example, the “nice” transcendent experiences described in Student focus group 1 might be considered everyday, albeit with a particular essence because of being associated with such a significant place. Meanwhile Margarita described how the Virgin Mary helped her every day. However most of the transcendent experiences were clearly more exceptional. For example, Susan, Sean, John, Pauline, and Maria variously described “huge experiences”, “the one real experience” and “vivid” emotional experiences, as well as others whose accounts evoked considerable intensity. Waldron also classifies transcendent experiences into three categories. The first involves “high religious intuition” including visions and sounds, and might accord with accounts of the participants who felt heard and touched. The second involves a sense of unity with the divine, perhaps like that described in our Theme 1, “Communicating with the Virgin Mary and God”. Waldron’s ultimate level is the highest state of enlightenment, which our participants did not explicitly describe.

Likewise, the transcendent experiences we report are not as extreme as the extraordinary spiritual experiences that transcend both ego and spatio-temporal boundaries, such as miraculous cures, out-of-body experiences and near-death experiences (Gesler, 1996; Underwood & Teresi, 2002). Participants did not claim these to be experiences of the kind that might be submitted to the Medical Bureau, and did not use terms like “miraculous”. The recognised miraculous cures arguably occupy a different conceptual space; exclusively physiological and therefore a case apart (Gesler, 1996). Notermans believes that “the dominant official discourse hardly recognises people's miraculous experiences” (Notermans & Jansen, 2011). According to the Medical Bureau, official miracles do occur in Lourdes, yet for such a tiny minority of visitors that they can scarcely be the focus for visitors or administrators, and they were not a focus among our participants.

A barrier to classifying experiences is the pilgrims’ struggles to express them. They repeatedly told us that it was hard to define the “something” that happened to them at Lourdes, although they described powerful effects. As Higgins and Hamilton describe in their study: “[William] James captures the affective power of Lourdes, drawing attention to its sensory qualities that cannot be verbally communicated” (Higgins & Hamilton, 2019). Their Lourdes participants described experiences where “something” highly significant, albeit not miraculous, happened to them (Higgins & Hamilton, 2014). This echoes James, Waldron and Cardeña et al., for whom such experiences are ineffable yet full of significance (Cardeña et al., 2014; James, 1902; Waldron, 1998).

One way to understand the intensity of transcendent experiences might be to explore their long term effects. For the most part our participants did not describe long term alterations to their lives or their illnesses as a result of transcendent experiences. However, several of the accounts related to an ongoing experience each time someone visited the Grotto, or to a memorable experience years ago, both of which suggest a lingering effect. Religious experience and religious ritual, including pilgrimage, can of course have transformative effects on people (Glannon, 2004; Pieper & Van Uden, 1994). Interestingly, a study of over 500 pilgrims from the Netherlands surveyed participants before, immediately after and six months after their pilgrimage, and while there was evidence of a deepening of faith and reflection immediately afterwards, they found that six months later there was no evidence of lasting change or transformation in people’s faith or religious attributions (Pieper & Van Uden, 1994). This recalls a common observation that people return regularly to pilgrimage sites for a “top-up” or renewal (Gesler, 1996; Higgins & Hamilton, 2019; Perriam, 2015). Of course, pilgrims may experience other kinds of transformation, relating to their world view or their perceptions of illness, for example, and these more subtle effects can be hard to detect in large surveys. Within therapeutic landscapes literature, Bell notes a lack of research exploring temporary respite as opposed to longer term transformation (Bell et al., 2018), and this could be a worthwhile question for future qualitative research with pilgrims. The simple facts of our participants describing a deepening of their faith and of recurring experiences at Lourdes hint at longer term effects.

Among the effects discussed were positive emotions in the moment, from milder feelings like contentment and peace through to ecstatic joy accompanied by tears. The importance of emotions among pilgrims to Lourdes has often been overlooked (Higgins & Hamilton, 2019), yet these positive emotions have potential health benefits. There is a growing body of evidence from positive psychology linking positive emotions with resilience (Fredrickson et al., 2003), psychosocial flourishing (Fredrickson & Losada, 2005), longevity (Moskowitz et al., 2008; Ostir et al., 2000) and reduced morbidity in several chronic diseases (Pressman et al., 2019). Others have shown that everyday spiritual experiences influence both positive and negative emotional states, enhancing positive affect while buffering the extent of negative affect (Whitehead & Bergeman, 2012). Deepening faith may have health implications too; Fisher asserts that spiritual well-being is an important component of general well-being, and a core aspect of spiritual well-being is the strength of relationships with “transcendent Others” (Fisher, 2010), like the closeness people feel with the divine in our data. We would expect these experiences to affect the relationship with the transcendent, but they also affect the relationship with the self, by bringing people closer to who they want to be. As our participant Pauline explained, “God really purified me and brought me to [be] a better person”. For many participants, transcendent experiences gave them a strong sense of affirmation in their faith. This confirmation from God or the Virgin Mary that they were watched, heard and guided echoed James’ “illuminations” which are inarticulate yet carry a sense of authority (James, 1902).

### The Significance of Place

Our participants’ experiences were contingent on their having taken place in Lourdes. It is well known as a therapeutic landscape, where physical, social and human conditions act together to promote healing, and it formed the basis for Gesler’s influential work (Gesler, 1996). What our interviews highlight is the very specific role of the Grotto, acknowledged time after time by our participants. It is arguably the core experience at Lourdes: in the study of 500 pilgrims, it was the only religious activity undertaken by every single person, from a list of 20 items (Pieper & Van Uden, 1994). We can consider its significance on several axes: from the everyday to the divine, mediated through Lourdes’ historical context; the natural and urban; and the interpersonal and intrapersonal.

Religious belief is inherently bound up with the significance of the place, and the whole site of Lourdes can be perceived as a “thin space” (Agnew, 2019), a place where there is a close connection between the everyday and the divine, as our participant Lizzie hinted in her description of the barrier between heaven and Earth being “really, really thin” in Lourdes, and where the transcendent can be seen as having broken through into normal life. Agnew writes that “a significant factor in the pilgrimage experience is the ability to collapse the boundaries between the celestial and the mundane” (Agnew, 2019) and having spoken to Lourdes pilgrims about other Marian sites they had visited, he reports that this effect, this immediacy and proximity, is specific to Lourdes. Lourdes, in particular the Grotto, is a place where the faithful can glimpse paradise. It is highly auspicious to the faithful as the site where the Virgin Mary appeared, echoes of which were in the minds of several of our participants. This highlights the fundamental yet often overlooked role of history, reputation and belief (Perriam, 2015). The Grotto is an evocative space, a focus for meaning (Gesler, 1996) and one where history, dominant discourses and expectations play forcefully in the minds of pilgrims.

The Grotto remains a natural area within what has become a purposefully landscaped site for spiritual ritual, an urban greenspace. Gates and a defined walkway have been built across the front of the Grotto and rows of benches face it, and as Eade notes, there is pattern of town planning domesticating the wilderness at Lourdes (Eade, 2022). Yet it is a hushed area of the domain, linked in memory to the wilderness it was for Soubirous. Gesler writes: “As one approaches the Grotto, everything becomes gradually quieter until finally the only sounds are the rippling of the river and whispers among the pilgrims” (Gesler, 1996). Fisher believes that spiritual well-being is augmented by a relationship with the natural environment (Fisher, 2010), and the presence of water in urban environments is associated with positive experiences and emotional benefits (Völker & Kistemann, 2011, 2013). We also note that many of the accounts of significant moments at the Grotto took place at night. Darkness and light have symbolic meanings within Christianity, and for anyone, the intimacy of candlelight within the darkness can alter senses and elicit emotions, especially among visitors searching for authentic experiences (Chevrier, 2019). For participants who are normally housebound or who lead busy urban lives, the chance to sit in silence at the “third space” of the Grotto may offer a rare chance to commune with nature as well as with God.

Lourdes facilitates both interpersonal and intrapersonal closeness. We have written elsewhere about the powerful sense of community experienced among pilgrims: connecting with other people through shared narratives and experiences, through reciprocity, through nourishing exchanges, is often a formative part of the Lourdes experience (Goldingay et al., 2021). However, it is the relationship with the self which often comes to the fore in these accounts of transcendent experiences. Several people touched on a sense of therapeutic listening, and participants talked about becoming better or truer versions of themselves, furthering this idea. Harris has likened the Grotto to the counsellor’s consultation room: an empathetic space where emotions can safely be aired and where one feels listened to (Harris, 2013). Perhaps Lourdes also provides a thin space between the current self and possible alternative versions of oneself. Both James and Fisher intimate that transcendent experiences are a part of who we are, and for the faithful, places like the Grotto allow this part of the self to surface more easily and be talked about more openly.

The image of pilgrims at the Grotto provides a metaphor to illustrate the diverse relational forces of this therapeutic landscape. Here we can envisage the liminal space between the physical and the heavenly—the “thin space” where pilgrims experience the presence of the divine, always aware of the site’s powerful history of collapsing the boundary between heaven and Earth. We see the close juxtaposition of the natural and urban elements, complementing each other and making the Grotto natural yet accessible. And we see the relationship between the individual and the community, the pilgrims sitting together in silent, candle-lit prayer; and perhaps between the individual and their other possible selves, brought into focus by divine communication. For the faithful, these webs of meaning can create an atmosphere conducive not only to well-being, but to transcendent experiences.

### Strengths and Weaknesses of the Study

One of the major strengths of this study is that it was carried out by an interdisciplinary team, and five individuals collected data. Another strength is the diversity of the subjects involved, who represented the many different backgrounds of those who visit Lourdes and the many different reasons for their coming. In addition, the data were varied, involving interviews, paired interviews and focus groups. An important limitation is that our fieldwork was restricted to English-speaking pilgrims: interviews with people of other nationalities might provide different articulations and experiences. Pilgrimage groups have notoriously full schedules and our fieldwork necessarily took place in the gaps; even so, we were able to gather data of considerable depth and breadth. We explicitly asked about special or unusual experiences to explore the question arising from our own earlier work. Another limitation is that the participants who volunteered may have been the more zealous or engaged pilgrims, and we could have sought more disparate voices. Similarly, by focussing on pilgrimage groups, we may have picked up dominant narratives from within those groups. Our fieldwork and analyses were carried out before the advent of COVID-19 and national lockdowns. These reduced pilgrim numbers by up to 95% in 2020, with older pilgrims the least likely to visit and some services offered online (Mróz, 2021). Precautions relating to COVID-19 may continue to be necessary in the years to come and may affect many aspects of the Lourdes experience, including those described here.

## Conclusions

Visiting Lourdes can have a powerful effect on a pilgrim and may include an “out of the ordinary” transcendent experience, involving a sense of relationship with the divine, or experiences of something otherworldly and intangible. There is a growing focus on Lourdes as a place with therapeutic benefits rather that cures: our analysis suggests that transcendent experiences can be central to this therapeutic effect. Such experiences can result in powerful emotional responses, which themselves may contribute to long term well-being. Our participants described a range of transcendent experiences, from the prosaic and mildly pleasant, to intense experiences that affected pilgrims’ lives. The place itself is crucially important, above all the Grotto, as a space where pilgrims perceive that the divine can break through into normal life, enabling closer connections with the divine, with nature and with the self.
